# Improper Remodeling of Organelles Deputed to Ca^2+^ Handling and Aerobic ATP Production Underlies Muscle Dysfunction in Ageing

**DOI:** 10.3390/ijms22126195

**Published:** 2021-06-08

**Authors:** Feliciano Protasi, Laura Pietrangelo, Simona Boncompagni

**Affiliations:** 1 CAST, Center for Advanced Studies and Technology, University G. d’Annunzio of Chieti-Pescara, I-66100 Chieti, Italy; laura.pietrangelo@unich.it (L.P.); simona.boncompagni@unich.it (S.B.); 2 DMSI, Department of Medicine and Aging Sciences, University G. d’Annunzio of Chieti-Pescara, I-66100 Chieti, Italy; 3 DNICS, Department of Neuroscience and Clinical Sciences, University G. d’Annunzio of Chieti-Pescara, I-66100 Chieti, Italy

**Keywords:** Ca^2+^ entry unit (CEU), Ca^2+^ release unit (CRU), excitation–contraction (EC) coupling, mitochondria, sarcoplasmic-reticulum (SR), store-operated Ca^2+^ entry (SOCE), transverse tubule (TT)

## Abstract

Proper skeletal muscle function is controlled by intracellular Ca^2+^ concentration and by efficient production of energy (ATP), which, in turn, depend on: (a) the release and re-uptake of Ca^2+^ from sarcoplasmic-reticulum (SR) during excitation–contraction (EC) coupling, which controls the contraction and relaxation of sarcomeres; (b) the uptake of Ca^2+^ into the mitochondrial matrix, which stimulates aerobic ATP production; and finally (c) the entry of Ca^2+^ from the extracellular space via store-operated Ca^2+^ entry (SOCE), a mechanism that is important to limit/delay muscle fatigue. Abnormalities in Ca^2+^ handling underlie many physio-pathological conditions, including dysfunction in ageing. The specific focus of this review is to discuss the importance of the proper architecture of organelles and membrane systems involved in the mechanisms introduced above for the correct skeletal muscle function. We reviewed the existing literature about EC coupling, mitochondrial Ca^2+^ uptake, SOCE and about the structural membranes and organelles deputed to those functions and finally, we summarized the data collected in different, but complementary, projects studying changes caused by denervation and ageing to the structure and positioning of those organelles: a. denervation of muscle fibers—an event that contributes, to some degree, to muscle loss in ageing (known as sarcopenia)—causes misplacement and damage: (i) of membrane structures involved in EC coupling (calcium release units, CRUs) and (ii) of the mitochondrial network; b. sedentary ageing causes partial disarray/damage of CRUs and of calcium entry units (CEUs, structures involved in SOCE) and loss/misplacement of mitochondria; c. functional electrical stimulation (FES) and regular exercise promote the rescue/maintenance of the proper architecture of CRUs, CEUs, and of mitochondria in both denervation and ageing. All these structural changes were accompanied by related functional changes, i.e., loss/decay in function caused by denervation and ageing, and improved function following FES or exercise. These data suggest that the integrity and proper disposition of intracellular organelles deputed to Ca^2+^ handling and aerobic generation of ATP is challenged by inactivity (or reduced activity); modifications in the architecture of these intracellular membrane systems may contribute to muscle dysfunction in ageing and sarcopenia.

## 1. Ca^2+^ Handling in Skeletal Muscle Fibers

**Figure 1:**
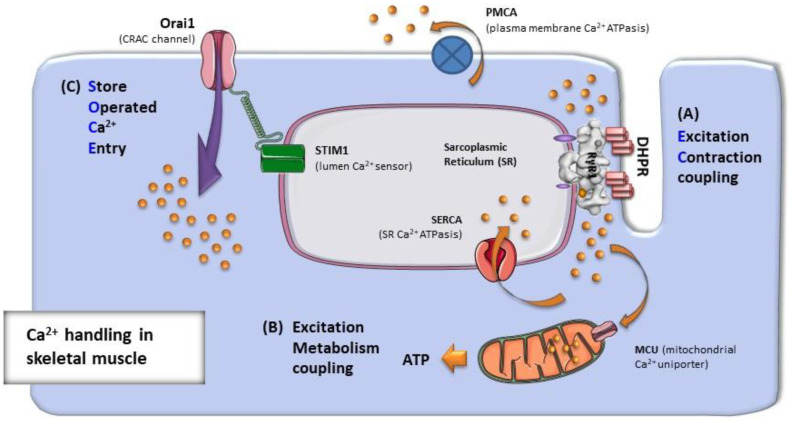
Ca^2+^ handling in skeletal muscle fibers. Intracellular Ca^2+^ concentrations in muscle fibers depend on: (**A**) the release and re-uptake of Ca^2+^ from intracellular SR stores during EC coupling, which controls the contraction and relaxation of sarcomeres; (**B**) the uptake of Ca^2+^ into the mitochondrial matrix during excitation–metabolism coupling, which stimulates aerobic ATP production; and finally (**C**) the entry of Ca^2+^ from the extracellular space via SOCE, a mechanism that is important to limit/delay muscle fatigue.

Calcium ion (Ca^2+^) is an extremely versatile intracellular messenger that plays an important role in all cell types and in a variety of physiological functions. Transient elevations in intracellular Ca^2+^ concentration ([Ca^2+^]_i_) indeed serve as rapid signals to regulate cell communication, gene transcription, differentiation, metabolic regulation, neurotransmitter release, etc. [1,2,3,4]. In striated muscles, myofibril contraction and relaxation are controlled by Ca^2+^ release and re-uptake from the sarcoplasmic-reticulum (SR). Ca^2+^ release follows the depolarization of exterior membranes (sarcolemma and transverse tubules or TTs): the mechanism that links sarcolemmal depolarization to Ca^2+^ release from the SR is known as *excitation–contraction (EC) coupling* (Figure 1) [5,6,7,8,9]. The relaxation of muscle fibers after each cycle of contraction (either single twitch or tetanic) is achieved mainly by the action of specialized SR proteins known as SERCA-pumps (sarco-endoplasmic reticulum Ca^2+^ ATP-asis), which quickly reduces myoplasmic [Ca^2+^]_i_ [10,11,12,13,14]. However, not all Ca^2+^ released during EC coupling is re-uptaken by the SR. A small amount of Ca^2+^ also enters the mitochondrial matrix to activate the mitochondrial respiratory chain and increase aerobic ATP production [15,16,17,18,19]: the effect of Ca^2+^ on the mitochondrial activity has been described as *excitation–metabolism coupling* (Figure 1). In addition, during repetitive and prolonged muscle stimulation, some Ca^2+^ is extruded in the extracellular matrix by plasma membrane (PM) Ca^2+^ ATPases (PMCA), causing a slight decrease in the SR Ca^2+^ content, a phenomenon known as *SR depletion* [20]. It has been demonstrated that SR depletion is the main trigger for activation of a mechanism known as *store operated Ca^2+^ entry (SOCE)* (Figure 1), which allows recovery of extracellular Ca^2+^ and replenishment of intracellular stores to counteract, or at least to limit, muscle fatigue [21,22,23,24,25,26].

### 1.1. Excitation–Contraction (EC) Coupling

The term EC coupling was coined in 1952 by Alexander Sandow [6,7,27] to describe the physiological series of events that convert the electrical stimulus that propagates on external membranes of muscle cells (i.e., the action potential) into a mechanical response of muscle fibers due to the activation of contractile elements [9,28,29,30,31]. Before knowing anything about the molecular machinery involved in EC coupling, it was already shown that Ca^2+^ was the ion activating contraction of muscle cells [9,28,29,30,31,32,33,34], that Ca^2+^ activating contractile elements was released from the SR [35,36], and finally that relaxation of fibers occurred thanks to its re-uptake into the SR [13,37,38].

EC coupling in mammalian muscle cells has evolved in two main mechanisms: (a) *Ca^2+^-induced Ca^2+^ release* (CICR), which is used by cardiac and smooth muscle to link entry of Ca^2+^ from the extracellular space to Ca^2+^ release from internal SR stores [39,40,41,42,43,44,45,46,47]; and (b) *mechanical coupling* of skeletal muscle [5,8,29,48], a direct communication that does not require external Ca^2+^ [36]. Both mechanisms use the same proteins (though different isoforms) to allow communication between external (sarcolemma or TTs) and internal membranes (SR): (a) voltage-gated L-type Ca^2+^ channels of external membranes, also known as dihydropyridine receptors (DHPRs); and (b) SR Ca^2+^ release channels, i.e., the ryanodine receptors (RYRs) [49,50,51,52,53,54,55,56].

The main difference between the two systems of communication is that the alpha-1c subunit of the cardiac DHPR (also known as Ca.V 1.2) functions as a voltage-gated Ca^2+^ channel allowing entry of external Ca^2+^ when sarcolemma and TTs are depolarized [47,57,58], in turn activating the RYR type-2 channel. Indeed, in cardiac cells, DHPRs are not directly linked to RYR2 subunits [59,60,61,62]. On the other hand, the term *mechanical coupling* in skeletal muscle was coined because the alpha-1s subunit (also known as Ca.V 1.1) of voltage-gated L-type Ca^2+^ channels in TTs acts as a voltage sensor which directly activates the SR Ca^2+^ release channels from RYR type-1 [5,8,29,48,54,63,64,65,66,67]. This direct communication is allowed by the formation of *tetrads*, groups of four DHPRs associated with the four subunits of RYR type-1 [68,69,70,71,72,73,74,75]. Thanks to tetrads, the signaling between DHPR and RYR1 subunits during EC coupling has been proposed to be bi-directional: *orthograde coupling* (DHPR to RYR1) allows DHPR voltage sensors (activated by depolarization of the TT membrane) to open RYR1 and activate Ca^2+^ release, while *retrograde coupling* (RYR1 to DHPR) allows RYR1 to influence the Ca^2+^ conductance and gating properties of DHPRs [76,77,78].

As the RYRs open following depolarization, either activated by Ca^2+^ entry (during CICR in cardiac cells) or mechanically by conformational changes of the DHPR voltage sensors (in skeletal fibers), Ca^2+^ released from the SR rapidly diffuses into the cytoplasm to generate *Ca^2+^ sparks*, i.e., confined rises in Ca^2+^ concentration first described in cardiomyocytes [79,80]. Ca^2+^ sparks were later detected also in amphibian striated muscle fibers [81], and in mammalian fibers, but only under special conditions such as after permeabilization by saponine, osmotic stress, membrane damage, etc. [82,83,84]. Contrary to the initial idea of one spark representing the opening of a single RYR, Shtifman et al. [85] estimated the number of release channels contributing to the generation of one event to be between 2 and 4. In the end, though, the near synchronous activation of thousands of Ca^2+^ sparks during an action potential causes a cell-wide increase in [Ca^2+^]_i_, known as *Ca^2+^ transient*. The Ca^2+^ released into the cytosol during EC coupling will finally bind to troponin C, a regulatory protein localized in the thin filament of sarcomeres, to activate the crossbridge cycling of myosin by removing the inhibition of tropomyosin [86,87,88]. Contraction will end when myoplasmic [Ca^2+^]_i_ will be lowered by removal, which is mainly operated by SERCA pumps [89,90].

Whereas DHPRs and RYRs are unquestionably the two main players in EC coupling, there are many other proteins that modulate and coordinate their interaction: calsequestrin, triadin, junctin, junctophilins, FK-506 binding protein-12 (FKBP-12), STAC3, histidine rich Ca^2+^ (HRC) binding protein, mitsugumins, etc. [91,92,93,94,95,96,97,98,99,100,101,102,103,104,105]. Most of those proteins are expressed in both cardiac and skeletal muscle cells (e.g., calsequestrin, triadin, junctin, junctophilins, junctate, FKBPs, HRC), while others are expressed only in one of the two (e.g., STAC3, JP45, and mitsugumins only in skeletal muscle). Together with RYR and DHPR, all those proteins constitute a macromolecular complex that coordinates the activation of SR Ca^2+^ release in skeletal fibers and cardiac cells.

### 1.2. Excitation–Metabolism Coupling

ATP is the source of energy for muscle fibers: (a) allows dissociation of the myosin heads from actin and its hydrolysis produces energy for force generation during the power stroke; (b) drives Ca^2+^ re-uptake by SERCA pumps during relaxation [106]; (c) is used by Na^+^/K^+^ ATPases to re-establish the proper balance of Na^+^ and K^+^ following the propagation of action potentials. As ATP is continuously used, several mechanisms are available to adequately match its availability: ATP may be produced anaerobically via hydrolysis of phosphocreatine, by anaerobic glycolysis [107,108], or by the aerobic metabolism within the mitochondrial matrix, which represents the major source of cellular ATP production in muscle [109]. A bi-directional interaction has been proposed between the SR and mitochondria in adult skeletal muscle fibers. Indeed this interaction involves an orthograde (SR to mitochondrion) and a retrograde (mitochondrion to SR) communication: (a) SR–mitochondrial signaling enhances aerobic ATP production through Ca^2+^-influx in the mitochondrial matrix [110,111,112]; whereas (b) mitochondrial-SR signaling would determine the ROS (reactive oxygen species)-dependent suppression of SR Ca^2+^ release [83,113].

Mitochondrial respiration and ATP production during muscle activity is controlled by a number of mechanisms [110,114]. Importantly, several mitochondrial dehydrogenases responsible for generating NADH (pyruvate, isocitrate, and 2-oxoglutarate dehydrogenases) and the ATP synthetic capacity of the F_1_F_0_-ATPase are stimulated by elevations in [Ca^2+^] in the mitochondrial matrix [110,111,114,115]. Thus, mitochondrial Ca^2+^ uptake during EC coupling (i.e., excitation–metabolism coupling) will stimulate aerobic ATP production to help keep pace with the increased ATP consumption associated with the augmented crossbridge cycling and the need for SERCA-mediated Ca^2+^ sequestration during muscle activity. An electrochemical gradient (>−180 mV) represents the driving force for mitochondrial Ca^2+^ entry. Several different mechanisms were postulated for entry of Ca^2+^ into the mitochondria: (i) a ruthenium red sensitive uniporter [116], (ii) a rapid mode Ca^2+^ transport mechanism [117], and (iii) a mitochondrial ryanodine receptor [118]. However, the recent identification of a mitochondrial calcium uniporter (MCU), a highly selective channel responsible for Ca^2+^ entry into mitochondria, may have unmasked the main pathway for mitochondrial Ca^2+^ uptake [119,120], even if the genetic ablation of MCU in the germline surprisingly displayed a mild phenotype [121].

The physiological relevance of mitochondrial Ca^2+^ uptake in skeletal fibers has long been debated, as the concentration of Ca^2+^ required for mitochondrial Ca^2+^ transport is considerably higher than the one achieved during global cytosolic Ca^2+^ transients elicited by EC coupling (~1–2 μM) [122,123,124]. A characteristic feature of the uniporter is its low affinity for Ca^2+^, with a Kd of around 10 μM in permeabilized cells [122,123,124]. MCU drives rapid and massive calcium entry, but only at cytosolic Ca^2+^ concentrations above the micromolar level (>10 μM) [123]. Nevertheless, elegant studies directly measuring mitochondrial Ca^2+^ clearly demonstrated a significant mitochondrial Ca^2+^ uptake in intact skeletal muscle [15,17,18,19]. Some studies proposed that this uptake occurs during both single twitches and tetanic stimulation [18], but these findings were not confirmed by others [125]. The apparent discrepancy between the low affinity mitochondrial Ca^2+^ transport and the magnitude of the Ca^2+^ transients could be reconciled by the concept of local Ca^2+^ microdomains (or nanodomains): strategic positioning of mitochondria in close proximity to sites of Ca^2+^ release would allow mitochondria to be exposed to a high pulse of Ca^2+^ before passive diffusion into myofibrils. An intimate structural interaction between mitochondria and endoplasmic/sarcoplasmic reticulum (ER/SR) have been demonstrated in non-muscle cells [126,127], in cardiomyocytes [128,129], and finally in skeletal muscle fibers [130].

In 1997, an elegant study reported how contractile relaxation was slower in more glycolytic fibers that contain fewer mitochondria than in mitochondria-rich slow twitch fibers [131]. It was argued that, although Ca^2+^ uptake by individual mitochondria is limited, a sufficiently large number of strategically positioned mitochondria may influence local and global Ca^2+^ transients elicited by EC coupling under physiological conditions. Other studies also demonstrated that the onset of local Ca^2+^ release events (Ca^2+^ sparks) in permeabilized skeletal muscle fibers was inversely proportional to the mitochondrial content, suggesting that mitochondria could inhibit spontaneous Ca^2+^ sparks [83,113]. Certainly, the evidence presented indicates that mitochondria are capable of physiological Ca^2+^ uptake, possibly during both single twitches and tetanic stimulation [16], though whether mitochondria accumulate significant amounts of Ca^2+^ and influence the size and temporal developments of Ca^2+^ transients remains a controversial issue.

### 1.3. Store-Operated Ca^2+^ Entry (SOCE)

During prolonged muscle activity a fraction of Ca^2+^ ions cycled by the SR during EC coupling is lost across the sarcolemma due to extrusion by Na^+^/Ca^2+^ exchangers of PM Ca^2+^ ATP-asis (PMCA) [132,133,134,135]. As loss of intracellular Ca^2+^, and a consequent reduction in the amount of Ca^2+^ stored in the SR (i.e., SR depletion), is one of the factors that may determine the onset of premature fatigue; muscle has developed a system to counteract this phenomenon and recover external Ca^2+^ during repetitive muscle activity [21,23,24,136,137,138]. This mechanism is known as SOCE, a pathway that allows extracellular Ca^2+^ to enter the cytosol and refill SR stores during muscle fatigue [138,139].

SOCE was first described in non-excitable cells, where its activation was triggered by the depletion of ER intracellular Ca^2+^ stores [20,140]. The molecular identity of the proteins responsible for SOCE, though, remained elusive for almost twenty years, until the two main proteins involved in this mechanism were discovered in patients affected by a severe immunodeficency: (a) stromal-interacting molecule-1 (STIM1), an ER protein which has an intraluminal portion that functions as a Ca^2+^ sensor [141,142,143]; and (b) ORAI1, a Ca^2+^ release activated channel (CRAC) of the PM [144,145,146,147]. The proposed mechanism for activation of SOCE involves depletion of intracellular stores that, in turn, induces Ca^2+^ dissociation from the luminal STIM1 N-terminal EF-hand domain and consequent conformational changes, dimerization, and relocation of STIM1 to ER sites of contact with external membranes [148,149,150]. Aggregated STIM1, in turn, recruits and traps ORAI1 channels into junctions, or *puncta*, formed by the association of STIM1-bearing ER with the external membrane. Finally, the STIM1-mediated opening of ORAI1 channels allows entry of Ca^2+^ into the cell from the extracellular space and replenishment of intracellular stores [151,152].

SOCE was reported in skeletal muscle in 2001 [24,137] when STIM1 and ORAI1 were not yet discovered. In 2008, though, Lyfenko and Dirksen [22] demonstrated that, also in skeletal muscle fibers, SOCE is mediated by interactions between STIM1, in the SR, and ORAI1 localized in the invaginations of the sarcolemma, the TTs, the specialized invaginations of the PM that carry the action potential [22,138,153]. In addition, calsequestrin-1 (CASQ1), a key SR protein that controls intracellular Ca^2+^ homeostasis [154] as a Ca^2+^ buffer and a modulator of RYR1, has been proposed to modulate SOCE [155,156,157]. SOCE in muscles developed some peculiarities with respect to other tissues, as a result of adaptation to the needs of skeletal fibers. For example, while, in non-muscle cells, the activation of CRAC channels needs over ~1 min [144], experiments in skinned skeletal muscle fibers have provided evidence that Ca^2+^ influx can be activated very quickly (<1 s) following Ca^2+^ store depletion [23,136,158]. One of the explanations, given a few years previously, for this rapid SOCE activation was that STIM1 and ORAI1 could be already clustered in pre-formed SR-TT junctions [25]. A *STIM1* splice variant highly expressed in skeletal muscle, *STIM1*-long, was discovered and proposed to mediate rapid SOCE activation [159,160]. There is now general agreement that SOCE plays an important role in muscle physiology not only as a mechanism to counteract fatigue, but also in other aspects of muscle functions from differentiation to early development and late life. Carrell and colleagues reported that *Orai-1* plays an important role in the maintenance of fatigue-resistant type I fibers [161]. In addition, impaired SOCE activity was proposed to contribute to muscle impairment in ageing [162,163,164], though this finding was not confirmed by others [165,166]. Finally, to explain the various roles of SOCE in skeletal muscle function, STIM1 has been proposed to act as a multipurpose stress transducer activated by different stimuli (depletion, oxidation, temperature, hypoxia and acidification) that, in turn, may regulate multiple downstream targets [167].

## 2. Architecture of the Membrane Systems and Organelles Involved in Ca^2+^ Handling and Aerobic ATP Production

In skeletal muscle fibers, the different functions that we described in Section 1 (i.e., EC coupling, cellular respiration, and SOCE) are executed by specific organelles: (a) EC coupling occurs at specialized junctions between SR and TT named Ca^2+^ release units (CRUs), or triads [9,168]; (b) aerobic ATP production takes place in mitochondria [169,170,171,172]; (c) SOCE is mediated by Ca^2+^ Entry Units (CEUs), again SR and TT junctions in continuity with membranes of CRUs, but with different morphologies and molecular components [173,174]. In adult fibers, each of these junctions/organelles retains a specific intracellular position, dictated somehow by the striation of contractile elements (see below). Cross striation is generated by the lateral alignment of contractile elements, long cylinders named myofibrils, which are constituted by repeating contractile units, the sarcomeres, in which thick and thin filaments generate alternating dark and pale bands—the A-band and the I-band, respectively.

### 2.1. Calcium Release Units (CRUs), or Triads: The Sites of EC Coupling

**Figure 2:**
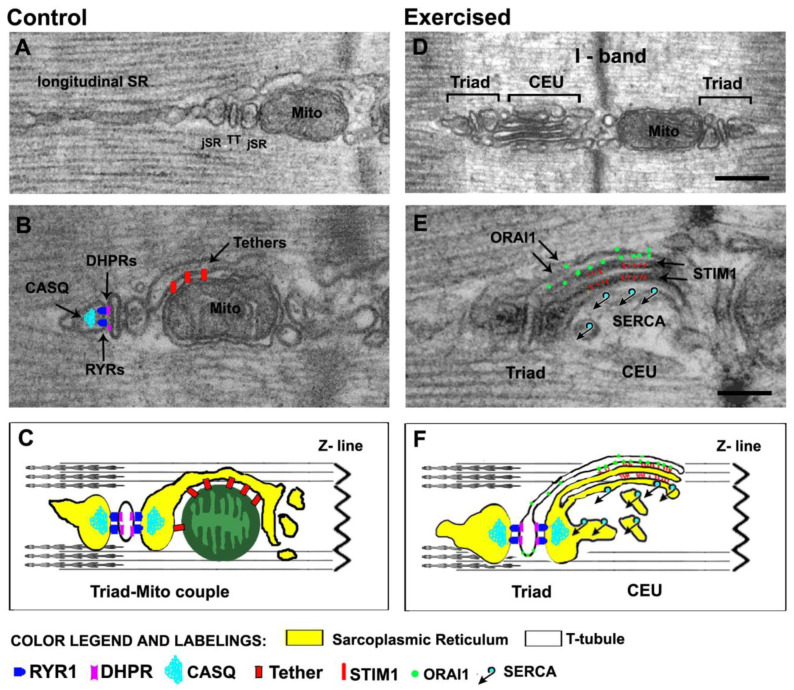
Architecture of the membrane systems and organelles involved in Ca^2+^ handling and aerobic ATP production. (**A**–**C**) In adult mammalian fibers, triads (or CRUs) are placed approximately at the A–I band transition when sarcomeres are relaxed. The SR that does not participate in the formation of junctions with TT constitutes the longitudinal SR, placed either at the A or I band. Triads contain the molecular players of EC coupling (RYRs, DHPRs, and CASQ) shown in panel (**B**) and are tethered to mitochondria, which are preferentially placed at the I band. In panel C, the cartoon shows a mitochondrion-triad couple. (**D**–**F**) Following acute exercise, sarcotubular membranes at the I band (both SR and TTs), are capable of significant remodeling, which eventually leads to the assembly of CEUs. CEUs are formed by SR-stacks, coupled with a TT and contain the molecular players of SOCE (STIM1 and ORAI1) shown in panel (**E**). In panel (**F**), the cartoon shows a triad and a CEU, two intracellular junctions which are structurally and molecularly different. Scale bars: (**D**) 0.2 μm; (**E**) 0.1 μm.

EC coupling in muscle fibers occurs in specialized structures, known as CRUs, formed by the close apposition of two membrane systems: TTs, carrying the sarcolemmal depolarization into the fiber interior [175], and the SR terminal cisternae, containing the Ca^2+^ needed for muscle activation [74]. CRUs in adult muscle are also named triads, as they are formed by three elements: a central TT—showing a narrow and flat profile—with two lateral terminal cisternae of the SR (Figure 2A,B) [73].

The assembly of triads in adult skeletal muscle fibers represents the final result of a differentiation-maturation process in which CRUs have different morphologies [73,74,176]. (Step 1) During differentiation, i.e., in myotubes, the interaction between DHPRs and RYRs occurs in junctions between the SR and sarcolemma, in peripheral couplings (PCs). (Step 2) Later, when fibers present a more organized contractile apparatus, and once the sarcolemma forms the invaginations known as TTs, the junctional SR (jSR) associates with longitudinal TTs, i.e., parallel to the long axis of myofibrils. (Step 3) Finally, in adult fibers, most TTs become transversal and assume their final position, approximately, at the transition between the I and A bands of relaxed sarcomeres (2.2–2.4 μm long), forming two transversal networks, one at each side of the Z-lines [177,178]. This specific position seems to be the final location of triads in mammalian fibers as it is retained both in fast and slow twitch fibers, with some difference in number/area and size of the individual elements [74,176].

The depolarization coming from the neuromuscular junction spreads along the sarcolemma where it is detected by the DHPRs, voltage sensors specifically localized in the TTs [8,54,179]. DHPRs are in direct communication with the closely apposed Ca^2+^ release channels of the SR, the RYRs (Figure 2B,C) [67,180,181,182]. The physical/mechanical coupling between DHPRs and RYRs occurs in triads and results in the sudden opening of the RYR Ca^2+^ release channels with a consequent massive efflux of Ca^2+^ from the SR lumen into the myoplasm [5,29,65,75] (see Section 1 for additional details). The observation of triads at higher magnifications using electron microscopy (EM) makes it possible to appreciate other important details:

(a) The gap between TT and SR is visually occupied by dark electron-densities, representing the cytoplasmic domain of RYRs, known as *feet*. RYR-feet in skeletal CRUs form ordered arrays in which RYRs touch each other corner-to-corner, forming ordered arrays [72,183].
(b) Inside the lumen of SR terminal cisternae, a dark matrix reveals the presence of calsequestrin (CASQ), the main SR Ca^2+^ buffer, which accumulates large amounts of Ca^2+^ in proximity of the sites of release [154,184,185,186,187,188]. The CASQ matrix appears to be anchored at the SR terminal cisterna via thin and long strands, which have been proposed to be constituted by triadin (Figure 2B,C) [189,190,191].

Contrary to RYRs feet, DHPRs voltage sensors are not visible in standard EM as they are almost entirely embedded within the TT lipid bilayer. DHPRs have, however, been visualized as peculiar groups of four particles named *tetrads*, in a series of elegant papers by C. Franzini-Armstrong and colleagues, through the use of freeze fracture (FF) preparations, a technique that makes it possible to break the lipid bilayer of TTs [61,72,75]. The demonstration that DHPRs need association with RYR1 subunits to form tetrads came in experiments in which lack of RYR1 in dyspedic cells resulted in DHPRs being randomly disposed, as in cardiac cells [70,71]. Re-expression of *Ryr1*, but not of *Ryr2* or *Ryr3*, two isoforms expressed mostly in cardiomyocytes and in the nervous system, restored tetrads’ arrangements [68,69].

### 2.2. Mitochondria: The Powerhouse of the Cell

In mammalian skeletal muscle fibers, myofibrils align laterally to generate a regular cross-striation, and sites of Ca^2+^ release occupy specific positions (see previous section). Interestingly, mitochondria, or at least a subpopulation of them, also seem to occupy a preferential disposition in skeletal muscle fibers. Ogata and Yamasaki, in EM studies, described a population of intermyofibrillar mitochondria surrounding myofibrils as incomplete rings in correspondence of the I band, on either side of the Z-line [192]. These I band mitochondria are present in fast, intermediate, and slow fibers of adult rat leg muscles. In addition to I-band mitochondria, slow-twitch fibers also contain large groups of mitochondria under the sarcolemma (mostly in proximity of capillaries) and often forming longitudinal columns between myofibrils, though columnar mitochondria are rare in fast-twitch fibers [192].

In 2009, we described the close proximity between mitochondria and sites of Ca^2+^ release [15,130], highlighting the presence of small electron-dense strands (generically named *tethers*) linking the outer mitochondrial membrane to the jSR (Figure 2A–C). Treatment with hypotonic solution, which caused swelling of jSR as well as mechanical stretching of tethers, was unable to disrupt the SR–mitochondria association [130]. However, despite these experiments suggesting that tethers may function as holders of the mitochondrial position next to CRUs, it is still unclear whether the EC coupling system is involved as a tether regulator. Even if a possible contribution of Mitofusin-2 in the formation was proposed [16], the molecular identity of tethers is still unknown.

The intimate association between mitochondria and CRUs is achieved progressively during post-natal maturation of skeletal fibers in a process that also involves remodeling of the mitochondrial network. Specifically, before reaching their final position next to triads (triadic mitochondria), the majority of mitochondria for a few weeks after birth (in mice), are disposed longitudinally between myofibrils and/or under the sarcolemma. This longitudinal disposition is similar to that frequently found in adult slow-twitch fibers, but rarely in fast-twitch fibers. Interestingly, the shift of the mitochondrial network from longitudinal to transversal closely mimics the maturation of the EC coupling apparatus development, except for a temporal delay, as TTs are completely transversal when most mitochondria are still longitudinal.

### 2.3. Calcium Entry Units (CEUs): The Dynamic Junctions That Mediate SOCE

Activation of SOCE requires an interaction between ORAI1, a CRAC channel placed in external membranes (either PM or TT) and STIM1, a Ca^2+^ sensor of ER/SR membranes [141,142,144,145,193,194,195]. In non-muscle cells, several studies proposed ER-PM junctions that form following the depletion of intracellular stores as the sites of SOCE [144,196,197]. Perni and colleagues, using FF preparations, also visualized clusters of ORAI1 channels in the puncta of HEK-293 cells [196].

While activation of SOCE in non-muscle cells requires several seconds, the kinetics of SOCE activation in skeletal muscle has been proposed to be faster [23,158,198]. This rapid SOCE activation suggested that STIM1–ORAI1 complexes should already be assembled, or at least pre-localized, in existing junctions [25]. As in triad junctions, TTs (external membranes which contain ORAI1) and SR (which contains STIM1) are already associated with each other to mediate EC coupling this site would have been, in principle, the perfect location for SOCE [23,25]. For the above reasons, the triad has been suggested as the ideal site for SOCE. Although the conclusion is not illogical, it ignores the fact that there is no direct evidence for the presence of STIM1/ORAI1 in triads. In addition, some peculiar features of the triad junctions may have represented a limit for proper STIM1–ORAI1 interaction; thus, the possibility that SOCE may occur at sites different from the triads remained open. The jSR–TT gap at the triad is a fairly crowded space where many proteins, such as RYR and DHPR, junctophilins, FKBP12, triadin, junctin, mitsugumin, STAC3, etc., modulate EC coupling and participate in the assembly and maintenance of the triad junction [70,168,191,199,200,201]. The presence of this complex macromolecular machinery may result in only limited opportunity for migrations of STIM1 oligomers in the SR to recruit ORAI1 channels in the TT.

Recently published evidence suggests that the most likely site for SOCE may indeed not be the triad [173,174,202,203,]. First, colocalization of STIM1 and ORAI1 is minimal in control conditions, with most of STIM1 placed in the SR at the I band [21,174], while ORAI1 is mostly placed in TTs at the triad junction, colocalized with an EC coupling protein (i.e., RYR1). Second, a single bout of incremental exercise on treadmill designed to induce muscle fatigue triggered a significant re-modelling of both SR and TT at the I band, and the formation of new junctions that are structurally and molecularly different from triads, as they do not contain RYRs, but colocalized STIM1 and ORAI1. (Figure 2D–F). STIM1–ORAI1 colocalization was promoted by elongation/relocation of TT bearing ORAI1 to the I band region rich in STIM1 [174,]. These new junctions were proposed to function as site of Ca^2+^ entry during SOCE, as their presence was accompanied by increased resistance of isolated muscles to fatigue in the presence of external Ca^2+^, but blocked by SOCE inhibitors 3,5-bis(trifluoromethyl)pyrazole derivative (BTP-2) and 2-aminoethoxydiphenyl borate (2-APB) [204,205]. These SR-TT junctions at the I band, named *Ca^2+^ Entry Units* (CEUs) [173,174], are also present in control conditions, just significantly smaller and less frequent, which could be the reason why they were never characterized and reported earlier. Whether these are the sites that mediate rapid SOCE in control conditions or if some STIM1–ORAI1 interaction occurs also in triads, is still debated, even if the former hypothesis is, in our eyes, the most likely. Additional strong evidence that junctions assembling during exercise at the I band are indeed sites of SOCE came when we reported that assembly of SR-TT junctions at the I band during SOCE resulted in increased rate of Mn^2+^ quench, in a time course study where TT elongation during exercise and retraction during recovery controlled the functional entry of divalent cations in the myoplasm of isolated single fibers [203]. Two other manuscripts finally showed: (a) that CEUs are constitutively assembled in CASQ1-knockout fibers [202], which undergo deep depletion during repetitive stimulation [206]; (b) that SOCE is dysfunctional in fibers of aged mice, where CEUs are reduced in number, but is rescued by long-term exercise [207]. A commentary published in *J. Gen. Physiol.* proposed CEUs as the backdoor for Ca^2+^ ions in muscle cells, bringing new attention to the importance of external Ca^2+^ in the function skeletal fibers [208].

## 3. Disarray of Intracellular Organelles in Denervation and Ageing

Ageing is a phenomenon which causes structural and functional deterioration of body organs. Among them, a significant decline in neuromuscular function and performance greatly affects quality of life for elderly individuals [209,210,211,212,213,214,215,216], as well as their independence in everyday life, with dramatic increases in health care costs [217,218,219]. One of the main effects of ageing is a drastic reduction in muscle mass that occurs in various measures in most individuals [220,221,222,223]. This phenomenon is known as *sarcopenia*, and causes a loss of 40 to 50% of muscle mass in sedentary individuals between the ages of 30 and 70 [223,224,225,226,227]. This severe muscle wasting is the combined result of a variety of changes: loss of motor units due to progressive denervation, fast-to-slow fiber type switching, mitochondrial loss, oxidative stress, etc. [211,228,229,230,231,232].

**Figure 3:**
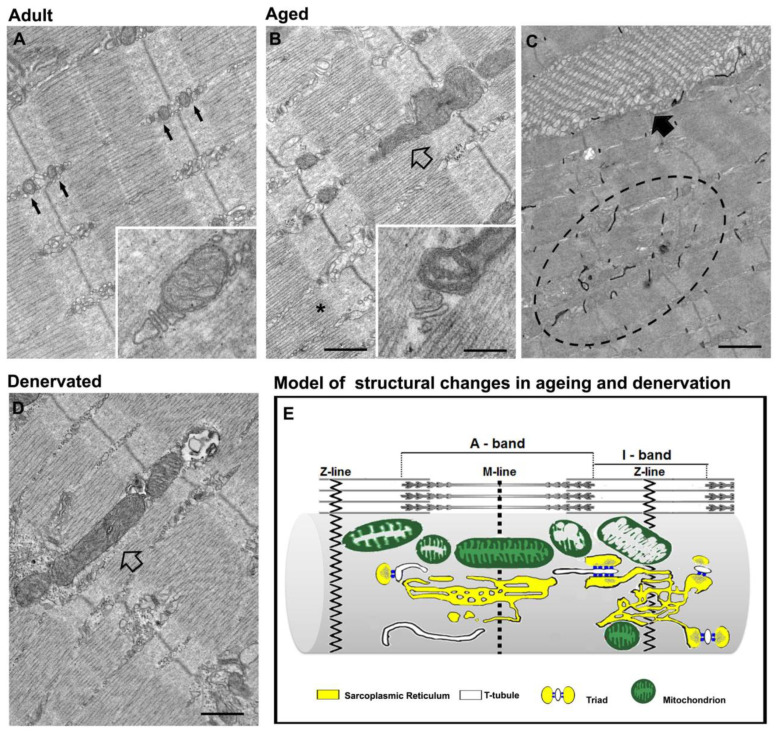
Disarray of intracellular organelles in denervation and ageing. **(A**–**D**) Representative EM images of EDL muscle fibers from adult (**A**), aged (**B**,**C**) and denervated mice (**D**). Ageing and denervation cause a similar misplacement and damage of mitochondria (**B**,**D**, empty arrows). In panel (**C**), a TA in ageing muscle (black arrow) and a disordered TT network (dashed oval), labelled in black with ferrocyanide, are shown. (**E**) A cartoon summarizing the main structural changes in ageing and denervation: loss of proper position of triads and mitochondria, and mitochondrial damage. Scale bar: (**A**,**B**,**D**) 0.5 μm; (**C**), 1 μm; insets, 0.1 μm.

In different, but complementary, projects we have collected several independent sets of data regarding the structural and functional decline of EC coupling and mitochondrial machineries caused either by long- and short-term denervation, or by sedentary ageing in human biopsies and animal models (rabbits, rats, and mice) (Figure 3). We have also collected some evidence of ultrastructural and functional changes occurring in the SOCE machinery of ageing mice.

### 3.1. Denervation Causes Disarray of EC Coupling and Mitochondrial Machineries

Among the numerous mechanisms that contribute to aged-related atrophy and degeneration of muscle tissue, denervation and loss of some motor units [233] seem to be important contributing factors. Several studies have shown that fast-twitch (type II) fibers are more affected by denervation than slow-twitch (type I) fibers [234,235] and that ageing leads to fast-to-slow transition in-fiber typing (thanks to cross innervation from other motor units), even if there is no general consensus on this finding.

Denervation of fast twitch motor units results in loss of muscle mass (atrophy), and functional and structural alterations of the fibers [236,237]. Between 2004 and 2019, we have studied the effect of short- and long-term denervation on muscle fibers in human biopsies from spinal cord injury (SCI) patients and in the muscle of rabbits and rats [238,239,240,241,242,243,244,245]. While a lot was known about the effect of denervation resulting in the reduction in the fiber diameter (atrophy) and in the disruption of contractile elements, its effect on the EC coupling system was completely overlooked. In two papers published in 2004 and 2007, we found that, following denervation, CRUs, which in adult mammalian fibers should be formed by three elements (i.e., triads; see Section 2 for additional details), lose proper orientation (becomes oblique or longitudinal), display altered morphology, and progressively disappear (their number is greatly reduced) [240,241]. This disarray of the EC coupling system was also confirmed in muscles from rabbits and rats, denervated for shorter periods (3–8 months) [243,244,245]. Interestingly, even after years of denervation, when fibers are only small tubes with a completely disrupted contractile apparatus, some deformed CRUs are still present [240,241]. The presence of some residual elements of the EC coupling machinery could explain why, even after 3–4 months of denervation, at least a subpopulation of muscle fibers maintains a partially functional EC coupling system [245]. It is worth noting that the changes in orientation, morphology, and number of CRUs detected in denervation resemble those of biopsies of elderly individuals (see next section below for additional details) [246].

Denervation in the muscles of rats and rabbits (3–8 months of denervation) also caused loss in proper disposition of mitochondria, which move from their preferential placement at the I band to become more longitudinal at the A band [243,244,245]. Interestingly, a similar pattern of de-modeling of the mitochondrial network was noted in muscle biopsies of elderly individuals and in extensor digitorum longus (EDL) muscle fibers of aged mice (see next section below for additional details) [247,248]. In Pietrangelo et al. 2019, we confirmed that denervation causes un-coupling of mitochondria from triads [238] and gathered some additional information about the timeframe causing these changes. Relocation of part of mitochondria in longitudinal columns between myofibrils is a relatively fast phenomenon, detected after 3–14 dd of denervation in mice, and after 15 dd of denervation in rats (Figure 3D). Moreover, it is worth noting that: (a) the loss of proper architecture of the metabolic machinery precedes the structural disarray of the EC coupling, suggesting that mitochondrial positioning is less stable than that of TT and SR (in Section 2, we described how, during post-natal maturation, the EC coupling system becomes transversal before the mitochondrial network; see also [130]); (b) the misplacement and un-coupling of mitochondria from CRUs is reversible and can be restored quite completely simply by muscle activity (an aspect that we will discuss more in depth in Section 4).

### 3.2. Misplacement of Intracellular Membranes and Organelles in Ageing

The diminished force output of ageing muscle is greater than the loss of mass, meaning that the *specific force* (SF) of ageing muscle (the force calculated by normalizing the maximal force produced by the muscle to its cross-sectional area) is smaller than the one measured in the adult. A possible explanation for the diminished SF could be a reduction in the supply of Ca^2+^ ions available for muscle contraction. An impairment in the events linking the action potential generated at the neuromuscular junction to the Ca^2+^ release from the SR has been proposed to explain age-related muscle weakness [229,249,250,251,252], and has been defined by Delbono and colleagues as *EC un-coupling* [250,253,254,255]. This un-coupling would be caused by a reduced expression/function of the α_1S_DHPR subunit, the voltage sensor of EC coupling in TTs (see Section 1.1). However, conflicting results can also be found in the literature, as a persistent expression of α_1S_DHPR in human ageing muscle was also reported [256].

To contribute to this controversy, we have studied the ultrastructure and geometry of the EC coupling (and of the mitochondrial) apparatus in human biopsies and murine muscles. Summaries of our results are given in the following paragraphs.

Boncompagni et al. 2006: contraction of muscle fibers is activated by a rapid increase in intracellular Ca^2+^ concentration known as Ca^2+^ transients. Transients, however, represent the final result of many individual Ca^2+^ release events, known as sparks [79,80,85], from different Ca^2+^ release sites [257,258,259], i.e. the triads, which in adult muscle are evenly distributed in the fiber interior, next to the I–A junction of each sarcomere (see Section 2.1. for additional details). To determine the possible reasons underlying the reduction in Ca^2+^ ions supply in ageing muscle [250,255], we analysed, using transmission EM, the ultrastructure and geometry of the EC coupling system in human muscle biopsies from vastus lateralis and glutaeus medius muscles of adult and aged individuals, and found that ageing causes significant disarray of membranes involved in EC coupling [246]. These alterations consisted primarily of: (a) progressive disarrangement of triads; and (b) reduction in the overall number of Ca^2+^ release sites available for triggering contraction. We reported a loss of 30–40% of release sites in aged specimens, a decrease that could explain the inefficient delivery of Ca^2+^ ions to the contractile apparatus and the impaired generation of force output. This finding provided a fairly sound structural explanation for the impaired transduction of the action potential into efficient increases in intracellular Ca^2+^ concentration reported in the literature [250,254].

Pietrangelo et al. 2015: in this study, we focused our attention on the association and functional crosstalk between CRUs and mitochondria using an integrated approach (not only EM, but also confocal microscopy, with functional and biochemical measurements of mitochondrial Ca^2+^ uptake, membrane potential and oxidative stress) [248]. This work reported: (a) that the n./volume of CRUs, mitochondria, and tethers decrease significantly with age, resulting in partial un-coupling between the two organelles; and (b) a fraction of mitochondria move from the I band position (Figure 3A) to being more longitudinally oriented at the A band (Figure 3B). Similar observations were also collected in human muscle biopsies from sedentary elderly individuals [247]. The age-related structural changes in number and disposition of CRUs and mitochondria with respect to myofibrils are shown in the cartoon in Figure 3E. The study also contains a correlation between structural modifications and functional changes, i.e., reduced Ca^2+^ transients and reduced mitochondrial Ca^2+^ uptake. The fact that these age-dependent changes are similar to those observed in human skeletal muscle biopsies [246,247] suggests that they may represent modifications that are specific to sedentary ageing. While mitochondrial modifications (structure, function, and number) have been widely reported both in age- and disease-related conditions [260,261,262,263,264], the data presented in this work draw attention to a previously unreported aspect: the fact that mitochondria tend to lose association to sites of Ca^2+^ release and position with respect to myofibrils.

Boncompagni et al. 2021: tubular aggregates (TAs) are abnormal accumulations of orderly disposed SR tubes which have been described in a variety of disorders [265,266,267,268,269,270], including TA myopathy (TAM), a disease recently linked to mutations in STIM1, ORAI1 [271,272,273,274,275,276], and CASQ1 [277]. Schiaffino et al. 1977 demonstrated that the formation of TAs is induced by anoxia in isolated rat muscle [278], and TAs have been also found in fast twitch fibers of male ageing mice (Figure 3C) [279,280,281]. In our knowledge, the presence of TAs has not been confirmed in aged human muscles.

In 2012, we showed how TAs, presumably representing improper remodeling of the SR, stain positive for CASQ1, but negative for RYRs, as triads are confined at their periphery [281]. In Boncompagni et al. 2021, TAs in EDL of aged mice were stained with antibodies against STIM1 and ORAI1, the two proteins that mediate SOCE: accumulation of STIM1 and ORAI1 in TAs correlates with an increased fatigability compared to adult mice [207]. Experiments in which extracellular Ca^2+^ was removed, unmasked the fact that muscles containing TAs seemed unable to use extracellular Ca^2+^ via SOCE during fatigue protocols [207]. These findings support previous works showing how a reduction in SOCE activity contributes to muscle weakness during ageing [162,163,164]. All of the above, together with the following additional two observations—(a) TAs accumulates, in addition to STIM1 and ORAI1, also CASQ1 [281], another protein that modulates SOCE [144,193,282], and (b) the presence of CEUs, the junctions that mediate SOCE, is reduced in aged fibers—led to the following conclusion: TAs must represent an SR demodeling that results in improper accumulation of proteins and dysfunctional SOCE.

## 4. Are Alterations in Denervation and Ageing Mainly Caused by Inactivity?

As described in detail in Section 2, both denervation and sedentary ageing result in severe disarray to EC coupling, mitochondrial, and SOCE machineries. However, in denervation, the injury causing muscle paralysis is not a direct insult to the muscle itself; hence, muscle should be, in principle, perfectly functional if appropriately stimulated. In ageing, on the other hand, one of the factors that needs to be taken into consideration is the progressive reduction in activity associated with ageing for both humans and animals. Hence, whether the alterations described in Section 3 are induced by denervation or by ageing per-se, or whether a lack of (or simply reduced) muscle activity would play a central role, remains to be determined.

### 4.1. Functional Electrical Stimulation (FES) Rescues the Ultrastructure of the EC Coupling Apparatus

We studied the effect of FES in different conditions: (a) in muscle of patients with SCI to restore muscle structure and mass; (b) as a supportive measure to improve muscle function in elderly individuals; and finally (c) as a therapy in a patient affected by a rare myopathy (central core disease, CCD) caused by a mutation in a protein involved in EC coupling (RYR1), and that causes mitochondrial damage and muscle weakness.

In a series of papers published between 2004 and 2010 [240,241,283,284], we used transmission EM to analyze muscle biopsies from patients who suffered complete lesion of the spinal cord. Their muscles were stimulated with electrical devices for prolonged periods (several years), but they started therapy at a time point in which alteration of muscle fibers due to denervation was already quite severe (a year or more). EM analysis of biopsies after therapy revealed a striking restoration of denervated muscles induced by home-based FES of those patients performing therapy 3–5 times a week [240,241,283,284]. The EM data published in Kern et al. 2004 and Boncompagni et al. 2007 [240,241] showed, beside FES-induced rescue of contractile elements, also rescue of the membrane elements that mediate EC coupling, with perfectly shaped triads placed in the correct sarcomeric position, i.e., at the transition between the I and the A bands. Retrospective analysis of EM micrographs also indicates that the mitochondrial position at the I band was rescued to some degree [130]. Experiments in denervated rabbits confirmed that FES is quite effective in rescuing the intracellular structure of skeletal fibers [244].

The mechanism underlying the recovery of skeletal fibers induced by FES in the absence of innervation continues to be debated. In Biral et al. 2008, we reported the presence of apparently atrophy-resistant fibers in permanently denervated human skeletal muscle [242]. In Squecco et al. 2009, experiments in denervated muscle from rats indicated that excitably of skeletal fibers persisted for prolonged periods after the denervation injury [245]. In Kern et al. 2004 and 2010, and in Boncompagni et al. 2007, we showed that some EC coupling units (i.e., triads), while deformed, are still present in muscle fibers even after years of denervation [240,241,284]. Finally, in Carraro et al. 2015, we reported how muscle fiber regeneration persists for years in long-term denervated muscle [285]. Altogether, these observations may provide the framework for the positive response of long-term denervated muscle to FES.

We also applied FES to different subjects or patients affected by muscle weakness:

1. As a supportive measure to improve muscle function in healthy, but sedentary, seniors: in this study, FES was able to improve the muscle trophism, force, and cross-sectional area of fast muscle fibers thanks to the up-regulation of IGF-1 and the down-regulation of some atrogenes (atrogin and MuRF-1) [286];
2. As an alternative therapy to improve muscle function and structure in a patient affected by a debilitating myopathy caused by a mutations in *RYR1* (i.e., central core disease, CCD) [287].

### 4.2. Exercise Prevents/Rescues the Ultrastructural Modifications Caused by Inactive Ageing and Denervation

Recently, we studied the effect of long-term training on the function, structure and connectivity between EC coupling units and mitochondria in humans and mice, focusing on: (a) muscle biopsies from well-trained seniors (average of 70 years of age) and age-matched healthy controls; (b) EDL muscles from 2-year-old mice, either sedentary or trained for 1 year in wheel cages; and finally (c) short-term denervated and re-innervated EDL muscles from adult rats. In the first study, three groups of male participants were included: (i) a group of well-trained seniors (average of 70 years of age) who exercised regularly in the previous 30 years of their lives, (ii) age-matched healthy sedentary seniors, and finally (c) active young men (average of 27 years of age). The results collected in this study [247] indicate that lifelong physical exercise prevented the age-related structural decay of intracellular organization, which causes misalignment of myofibrils, and partial misplacement of EC coupling and mitochondria from their proper intracellular disposition. Furthermore, the mitochondrial volume, n./area of mitochondria, and number of properly coupled mitochondria to triads in exercised individuals (average of 70 years) are approximately double those of age-matched healthy sedentary seniors, and similar to those of active young men (average of 27 years) [247].

Similar results were collected in a murine model in which WT mice were allowed to age for up to 24 months [238]. Two groups of WT mice were studied: sedentary aged mice (housed for 24 months in regular cages) and aged-trained mice (housed for the first 12 months in regular cages, and from 12 to 24 months of age in wheel cages for voluntary running). Long-term voluntary exercise prevented the ultrastructural decay of the EC coupling and mitochondrial machineries caused by sedentary ageing. In addition, long-term exercise rescued muscle function, and reduced oxidative stress, which was proposed in Pietrangelo et al. 2015 to cause damage to membranes and organelles deputed to Ca^2+^ handling and aerobic ATP production [248]. Physical exercise was also shown to: (a) increase the expression levels of the MCU and affect mitochondria dynamics [288], and (b) promote muscle reinnervation with age in ageing human skeletal muscle [289].

In Pietrangelo et al. 2019, we also studied short-term denervated EDL muscles from adult rats before (15 dd) and after (15 + 30 dd) sufficient time was given to the nerve to reinnervate muscle fibers [238]. These experiments showed how the misplacement of mitochondria at the A band (and consequent un-coupling from triads) resulting from short-term denervation (15 dd) was perfectly rescued by reinnervation (15 + 30 dd), suggesting that the rapid loss of mitochondrial position due to complete muscle inactivity is reversible.

Some preliminary findings have been also collected regarding the effect of inactivity and exercise on the membrane systems handling SOCE. SOCE in muscle fibers is handled by CEUs, junctions which are formed at the I band during exercise through the association of TTs and SR-stacks (see Section 2.3 for additional details). In Boncompagni et al. 2021, we showed how sedentary ageing in mice results in loss of SOCE function and reduced presence of the structures needed for the assembly of functional CEUs (TTs at the I band) [207]. A significant amount of dysfunctional STIM1 and ORAI1 was accumulated in TAs, representing peculiar assembly of SR tubes; this was found in ageing and in several myopathies. Importantly, the study also demonstrated that long-term training of mice in wheel cages (a) reduced the formation of TAs, (b) prevented the loss of CEUs, and (c) rescued functional SOCE [207].

## 5. Final Remarks

Adult skeletal muscle fibers are marvelous multinucleated machines designed to efficiently produce force while burning energy. Beside constituting the fundamental units deputed to movement, in the end—if they work properly—they may represent the metabolic balance of the whole organism. However, to work properly, skeletal fibers need a specific arrangement of the intracellular organelles and membranes deputed to force production (myofibrils), Ca^2+^ handling (CRUs and CEUs), and aerobic ATP production (mitochondria). This arrangement (discussed in detail in Section 2) is achieved during a process of differentiation and post-natal maturation that may take months or years depending on the species we are considering (e.g., mice vs. humans).

In this review, we have focused our attention on an aspect that is not often discussed and that has attracted our attention. In skeletal muscle fibers, intracellular ordered disposition of organelles is lost quite quickly when muscle activity is absent or reduced (we have studied denervation and sedentary ageing; see Section 3), suggesting that the preservation of proper architecture requires muscle activity. This hypothesis is supported by the studies in which we used FES and exercise to prevent or reverse the remodeling caused by inactivity. Changes caused by muscle inactivity are transversal (from humans to animal models) and somehow similar in ageing and early-denervation, though more severe in the latter, especially when denervation is prolonged.

The studies reviewed in Section 3 and Section 4 support the idea that the intracellular architecture of fibers is quite important for the proper function, both contractile and metabolic, of muscle. Indeed, the disarray of membranes involved in EC coupling (CRUs), excitation–metabolism coupling (close association CRU-mitochondrion), and SOCE (CEUs) may contribute to impaired delivery of Ca^2+^ and the production of ATP (required for optimal force generation by myofibrils), and to muscle fatigue, thus causing reduced muscle performance and dysfunction.

It is important to underline that the loss and misplacement of CRUs, mitochondria, and CEUs appears to be a reversible process, as it can be prevented or rescued by muscle activity/exercise (discussed in Section 4), a fact that emphasizes the great plasticity of muscle fibers in response to an appropriate stimulation.

## Abbreviations

Ca^2+^-induced Ca^2+^ release	CICR
calcium release unit	CRU
calcium entry unit	CEU
dihydropyridine receptor	DHPR
electron microscopy	EM
excitation–contraction	EC
functional electrical stimulation	FES
ryanodine receptor	RYR
sarcoplasmic-reticulum	SR
store-operated Ca^2+^ entry	SOCE
tubular aggregate	TA
transverse tubule	TT
